# Stem cells and exosomes: promising candidates for necrotizing enterocolitis therapy

**DOI:** 10.1186/s13287-021-02389-4

**Published:** 2021-06-05

**Authors:** Ruijie Zeng, Jinghua Wang, Zewei Zhuo, Yujun Luo, Weihong Sha, Hao Chen

**Affiliations:** 1 Department of Gastroenterology, Guangdong Provincial People’s Hospital, Guangdong Academy of Medical Sciences, Guangzhou, 510080 China; 2 Shantou University Medical College, Shantou, 515041 China; 3 Department of Hematology, Guangdong Provincial People’s Hospital, Guangdong Academy of Medical Sciences, Guangzhou, 510080 China

**Keywords:** Stem cell, Exosome, Breast milk, Necrotizing enterocolitis

## Abstract

Necrotizing enterocolitis (NEC) is a devastating disease predominately affecting neonates. Despite therapeutic advances, NEC remains the leading cause of mortality due to gastrointestinal conditions in neonates. Stem cells have been exploited in various diseases, and the application of different types of stem cells in the NEC therapy is explored in the past decade. However, stem cell transplantation possesses several deficiencies, and exosomes are considered potent alternatives. Exosomes, especially those derived from stem cells and breast milk, demonstrate beneficial effects for NEC both in vivo and in vitro and emerge as promising options for clinical practice. In this review, the function and therapeutic effects of stem cells and exosomes for NEC are investigated and summarized, which provide insights for the development and application of novel therapeutic strategies in pediatric diseases. Further elucidation of mechanisms, improvement in preparation, bioengineering, and administration, as well as rigorous clinical trials are warranted.

## Introduction

Necrotizing enterocolitis, a devastating disease predominately affecting neonates, has attracted wide attention of researchers for decades. As a leading cause of death among gastrointestinal diseases in neonates, NEC occurs in 1–3 per 1000 live births every year in the USA [1]. The mortality of infants suffering from NEC is estimated as 20 to 40%, while infants with less gestational ages demonstrate higher mortality [2]. In addition, the mortality of infants requiring NEC surgery can be up to 50% [2]. Despite treatment advances, the morbidity and mortality remain high. Short-term complications of NEC include short bowel syndrome, intestinal dysmotility, strictures, perforation, and sepsis, while intestinal failure, neurodevelopmental delay, and failure to thrive can be developed in the long term.

The etiology of NEC is multifactorial, and several risk factors that predispose neonates to NEC are identified. The best-known risk factors for NEC are low gestational age and low birth weight. In addition, formula feeding and prolonged parenteral feeding increase the risk of NEC. Congenital heart diseases, polycythemia, and respiratory distress are also reported as risk factors for NEC. Moreover, maternal conditions, including pregnancy-induced hypertension, preeclampsia, smoking, gestational diabetes, and chorioamnionitis, are reported to induce NEC [3].

For pathogenesis, the susceptibility and hyper-reactivity of preterm gut, which predispose the infantile intestine to altered immune response, vascular injury, and dysregulated microbiota, are the root causes contributing to NEC [4]. Intestinal mucosal hypoxia and impairment of the intestinal microcirculation are correlated with the development of NEC [5]. The pathoimmunological changes of NEC include deficiency in anti-inflammatory mediators and increases in pro-inflammatory mediators [6, 7]. The anti-inflammatory cytokine interleukin (IL)-37 and its receptor, as well as protective innate lymphoid cells are deficient in NEC, while pro-inflammatory innate lymphoid cells are increased [7]. Alterations of IL-37 also mediate the modulation of toll-like receptor (TLR) expression. The innate immune receptor TLR4 that specifically recognizes bacterial lipopolysaccharide (LPS) is upregulated in premature infant intestine and activates innate immunity [8]. TLR4 activation promotes inflammation by enhancing the activity of pro-inflammatory cytokines and signals, leading to the NEC development [7, 8]. Defective Wnt signaling in intestinal stem cells (ISCs) and impaired intestinal regeneration also contribute to NEC development [9]. However, specific mechanisms directly lead to NEC remain largely unknown. The lack of understanding of the pathogenetic mechanisms in depth restricts the development of NEC-specific treatment modalities.

The mainstays of NEC treatment include cessation of enteral feedings, gastric decompression, antibiotics, parenteral nutrition, and surgery, and they are non-specific. Despite therapeutic advances, severe NEC (Bell stage III) is often considered incurable. Therefore, novel treatment modalities are urgently needed for NEC therapy. Emerging strategies for the treatment of neonates with NEC have been explored by researchers in recent years, in which stem cells and exosomes demonstrate promising effects and potential clinical application.

In this review, the applications of stem cells and exosomes for the NEC therapy are investigated. Our review principally aims to integrate current studies on NEC therapy by stem cells and exosomes and therefore provides insights for the development and application of novel therapeutic strategies for NEC.

## Stem cells and extracellular vesicles

### Biology and function of stem cells

Stem cells are defined as unspecialized cells possessing the capacity of self-renewal and generation of multiple mature cell types [10]. Basically, stem cells can be classified based on origins or differentiation potential (Table 1). On the one hand, a simple and convenient way to categorize stem cells is to divide them according to their origins, for example, embryonic stem cell (ESC) and adult stem cell (ASC). Induced pluripotent stem cells (iPSCs) have emerged in recent years as the third type of stem cells. Compared to ESCs, ASCs are most successfully applied in human therapy, with the advantages of abundant resources, less ethical concerns and lower risk of rejection [11]. Further definition of ASCs can be based on their sources. On the other hand, the capacity of stem cells to differentiate into distinct cell types varies, and therefore stem cells can also be classified into unipotent, oligopotent, multipotent, pluripotent, and totipotent stem cells. ASCs are regarded as multipotent cells, indicating that they are able to differentiate into multiple cell types but restricted to certain lineages.

**Table 1:** Classifications of stem cells

Classification	Stem cell types	Definition	Examples
Origin	Embryonic	Stem cells derived from the early stages of embryos	/
	Adult	Stem cells identified throughout the body that divide to replenish dying cells and regenerate damaged tissues	
	Induced pluripotent	Stem cells derived from adult cells by being reprogrammed to embryonic stem cell-like states	
Differentiation potential	Unipotent	Stem cells with the capacity to differentiate into only one specific cell type	Muscle satellite cells
	Oligopotent	Stem cells with the capacity to differentiate into only a few cell types	Lymphoid stem cells
	Multipotent	Stem cells with the capacity to differentiate into multiple cell types	Adult stem cells
	Pluripotent	Stem cells with the capacity to differentiate into nearly all cell types	Embryonic stem cells
	Totipotent	Stem cells with the capacity to differentiate into all cell types and a functional organism	Zygotes

Intestinal epithelial cell lineages can be harmed by stressors such as ischemia, hypoxia, and hypothermia, especially in NEC, and ISCs are responsible to facilitate the repair and regeneration of intestinal epithelial cell lineages harmed by stressors in NEC [12]. Currently, two types of stem cells have been identified in small intestinal crypts: crypt base columnar cells and “+4” cells, which are cycling and quiescent, respectively [13]. CBC cells mainly express LGR5 and prominin-1, while “+4” cells principally express Bmi1 [13]. The characterization of LGR5 as a marker for mitotically active ISCs, which exhibit sensitivity to canonical Wnt modulation and contribute to homeostatic regeneration, allows researchers to study the function of ISCs in NEC.

The reduced proliferation of ISCs contributes NEC exacerbation, which indicates that maintaining the homeostasis of intestine and protecting ISCs within the newborn intestine are essential [9]. Heparin-binding epidermal growth factor (EGF)-like growth factor (HB-EGF), which was first identified in the conditioned medium of macrophage-like cells, can protect ISCs from in vitro hypoxic injury and in vivo NEC model [14]. In addition, all-trans retinoic acid, a vitamin A derivative, restores T cell balance, protects ISC pool, and further prevents the development of NEC [15]. Moreover, exogenous provision of Wnt can activate ISCs to promote the proliferation, differentiation, and repair of injured intestinal epithelium in experimental NEC, which indicates that Wnt/β-catenin signaling is the upstream pathway for ISC-modulated intestinal regeneration [9]. Although ISCs are originated from intestine, hurdles exist for ISC transplantation, which include incomplete understanding of human ISCs/progenitor and niche cells, difficult expansion in vitro, and insufficient methods to enrich ISC subtypes as well as undeveloped methods to integrate ISCs into existing intestinal mucosa [16]. Consequently, current studies mainly focus on the transplantation of other types of ASCs. The study of stem cell transplantation on inflammatory bowel disease is most advanced among all intestinal diseases. Guided by the successful experimental and clinical application of stem cells in inflammatory bowel diseases [17], which shares similar features with NEC, the study of stem cells on NEC is emerging in recent years.

In addition to the basic ability of regeneration and replacement, stem cells also act by paracrine actions. Under certain conditions, a variety of paracrine factors can be secreted by stem cells, which exert different biological effects such as anti-inflammation, anti-apoptosis, promoting proliferation, and enhancing migration [18].

### Biology and function of extracellular vesicles

Extracellular vesicles (EVs) are nanosized, cell-derived membrane-bound vesicles functioning as a means of cell to cell communication. A wide spectrum of cells, not limited to stem cells, can release EVs, which contain biological materials including DNA, RNA, lipids, and proteins, into biological fluids. Apoptotic bodies, microvesicles, and exosomes are the three main types of EVs. Apoptotic bodies (0.5–2.0 μm) are released as products of cell apoptosis. Microvesicles (0.1–1.0 μm) are vesicular structures budding directly from the plasma membrane. Exosomes (40–120 nm) are smaller vesicles formed by the fusion of multivesicular bodies and the plasma membrane [19]. Among the three types of EVs, exosomes are most studied and best characterized in various diseases.

Exosomes are extensively explored for their therapeutic potential in fundamental research, demonstrating novel capacities in treating intractable conditions. Several clinical trials have reported the application of exosomes, and most of them are studies for patients with cancers [20]. To date, there is no registered clinical trial of stem cells or exosomes for NEC. Our integration of current findings on fundamental and preclinical research might evoke the application of exosomes for NEC therapy clinically.

## Stem cells in necrotizing enterocolitis therapy (Table 2)

In the past two decades, stem cell therapy has become a heated topic for researchers and promising results are illustrated. The study of stem cells on NEC emerged approximately a decade ago, with a dramatic increase in number in the last few years.

**Table 2:** Functions and applications of stem cells in NEC

Stem cell type	Stem cell origin	Stem cell administration	Model	Modeling method^a^	Main findings	Mechanism	Reference
**Bone marrow-derived stem cell (BM-MSC)**							
BM-MSC	Human	Intraperitoneal injection	In vivo	Rat pups with formula feeding, hypoxia-hyperoxia (100% CO_2_/97% O_2_, 10 min/5 min, bid for 3 days) and hypothermia (4 °C, 5 min, bid for 3 days)	BM-MSCs administrated by intraperitoneal injection improve pathological changes of the neonatal NEC rat model. BM-MSCs injected rats show significant weight gains and clinical sickness score improvement.	/	[21]
BM-MSC	Mouse homozygotes	Intraperitoneal injection Intravenous injection	In vitro In vivo	Rat pups with hypoxia (100% N_2_, 1 min, bid until the end of experiment) and hypothermia (4 °C, 10 min, bid until the end of experiment)	BM-MSC proliferation, migration, and survival are increased by HB-EGF. BM-MSCs administrated intravenously have increased engraftment into intestine compared to BM-MSCs administrated intraperitoneally. BM-MSCs and HB-EGF co-administration significantly lowers the incidence of NEC and improves gut barrier function as well as survival.	/	[14]
BM-MSC	Human	Intravenous injection	In vivo	Preterm fetal sheep with umbilical cord occlusion (25 min)	BM-MSCs administrated intravenously do not relieve hypoxia-ischemia induced adverse intestinal events, which may be associated with NEC.	/	[22]
BM-MSC	Adult rat	Intraperitoneal injection	In vivo	Rat pups with formula, hypoxia (100% N_2_, 1.5 min, tid for 4 days) and hypothermia (4 °C, 10 min, tid for 4 days)	BM-MSC, AF-MSC, amniotic fluid-derived NSC and enteric NSC treatments all show a significant decrease in intestinal permeability and improved gut barrier function compared to the control group. There is no significant difference in intestinal permeability or gut barrier function among the four treatment groups.	/	[23]
BM-MSC	Adult rat	Intraperitoneal injection	In vivo	Rat pups with formula, hypoxia (100% N_2_, 1.5 min, tid for 4 days) and hypothermia (4 °C, 10 min, tid for 4 days)	BM-MSC, AF-MSC, amniotic fluid-derived NSC, and enteric NSC treatments all show significant reductions in NEC incidence compared to the control group. There is no significant difference in incidence among the four treatment groups.	/	[24]
BM-MSC	Adult rat	Intraperitoneal injection (conditioned medium)	In vitro In vivo	Rat pups with hyperosmolar formula, hypoxia (5% O_2_, 95% N_2_, 10 min, tid for 2 days) and LPS (4 mg/kg, qd for 2 days)	Condition medium of PHD2-silenced BM-MSCs repairs the intestinal damage and improves the survival of NEC rats. BM-MSCs’ paracrine effect is enhanced by PHD-2 silencing. PHD-2 silencing activates NF-κB and promotes IGF-1 as well as TGF-β2 secretion in BM-MSCs.	PHD2–NF-κB–IGF-1, TGF-β2	[25]
**Amniotic fluid-derived mesenchymal stem cell (AF-MSC)**							
AF-MSC	E14 rat	Intraperitoneal injection	In vivo	Rat pups with hyperosmolar formula, hypoxia (5% O_2_, 95% N_2_, 10 min, tid for 2 days) and LPS (4 mg/kg, qd for 2 days)	BM-MSCs lack beneficial effects on survival. AF-MSCs improve survival and increase the repair of injured intestine in NEC via a COX-2 dependent mechanism. AF-MSCs decrease bowel inflammation, increase cell proliferation and reduce cell apoptosis. AF-MSCs mediated effects do not depend on direct repopulation, but on a paracrine manner.	Stromal cells expressing COX-2	[26]
AF-MSC	E14 rat	Intraperitoneal injection	In vivo	Rat pups with hyperosmolar formula, hypoxia (5% O_2_, 95% N_2_, 10 min, tid for 2 days), and LPS (4 mg/kg, qd for 2 days)	AF-MSCs decrease fluid retention and lower the incidence of ascites in NEC rats.		[27]
AF-MSC	E14.5 rat	Intraperitoneal injection	In vivo	Rat pups with formula, hypoxia (100% N_2_, 1.5 min, tid for 4 days) and hypothermia (4 °C, 10 min, tid for 4 days)	BM-MSC, AF-MSC, amniotic fluid-derived NSC and enteric NSC treatments all show significant reductions in NEC incidence compared to the control group. There is no significant difference in incidence among the four treatment groups.	/	[24]
AF-MSC	E14.5 rat	Intraperitoneal injection	In vivo	Rat pups with formula, hypoxia (100% N_2_, 1.5 min, tid for 4 days) and hypothermia (4 °C, 10 min, tid for 4 days)	BM-MSC, AF-MSC, amniotic fluid-derived NSC, and enteric NSC treatments all show a significant decrease in intestinal permeability and improved gut barrier function compared to the control group. There is no significant difference in intestinal permeability or gut barrier function among the four treatment groups.	/	[23]
AF-MSC	Mouse pup	Intraperitoneal injection	In vivo Ex vivo	Mouse pup with formula, exposure to hypoxia for 4 days and oral LPS injection (4 mg/kg for 2 days)	AF-MSC rescues intestinal injury, restores epithelial regeneration, and increases active ISCs.	Wnt	[28]
**Umbilical cord-derived mesenchymal stem cells (UC-MSC)**							
UC-MSC	Human	Intraperitoneal injection	In vitro In vivo	Rat pups with formula gavage (supplemented with 8 mg/kg LPS), hypoxia (5% O_2_, 95% N_2_, 10 min, tid), and hypothermia (4 °C, 10 min, bid)	UC-MSCs exert beneficial effects in NEC via the production of the paracrine mediator H_2_S. UC-MSCs produce more H_2_S under hypoxic conditions.	H_2_S	[29]
**Neural stem cell (NSC)**							
NSC	Mouse embryos at 12.5 days post coitum	Intraperitoneal injection	In vivo	Rat pups with hypertonic formula, hypoxia (100% N_2_, 1 min, bid for 3 days), hypothermia (4 °C, 10 min, bid for 3 days), and LPS (2 mg/kg, 8 h after birth)	Myenteric plexus ganglia are damaged in NEC patients. NSC transplantation improves the enteric nervous system, intestinal integrity, stem cell differentiation, and intestinal transit, as well as decreases the mortality of NEC rats.	/	[30]
NSC	E11.5 mouse	Intraperitoneal injection	In vitro In vivo	Mouse pups with formula, hypoxia (100% N_2_, 1 min, bid for 3 days) and hypothermia (4 °C, 10 min, bid for 3 days)	NSC transplantation reduces NEC incidence. NSC injection improves gut barrier function and intestinal motility. NSC-HB-EGF co-administration or HB-EGF-overexpressed NSC has augmented therapeutic effects on NEC.	/	[31]
AF-NSC	E14.5 rat	Intraperitoneal injection	In vivo	Rat pups with formula, hypoxia (100% N_2_, 1.5 min, tid for 4 days) and hypothermia (4 °C, 10 min, tid for 4 days)	BM-MSC, AF-MSC, amniotic fluid-derived NSC, and enteric NSC treatments all show significant reductions in NEC incidence compared to the control group. There is no significant difference in incidence among the four treatment groups.	/	[24]
E-NSC	Rat pup						
AF-NSC	E14.5 rat	Intraperitoneal injection	In vivo	Rat pups with formula, hypoxia (100% N_2_, 1.5 min, tid for 4 days) and hypothermia (4 °C, 10 min, tid for 4 days)	BM-MSC, AF-MSC, amniotic fluid-derived NSC, and enteric NSC treatments all show a significant decrease in intestinal permeability and improved gut barrier function compared to the control group. There is no significant difference in intestinal permeability or gut barrier function among the four treatment groups.	/	[23]
E-NSC	Rat pup						
NSC	/	/	In vitro In vivo	Rat pups with formula, hypoxia (100% N_2_, 1 min, bid for 4 days) and hypothermia (4 °C, 10 min, bid for 4 days)	NSC differentiation in ENC rats is increased by HB-EGF. NSC expression of nNOS is enhanced by HB-EGF.	/	[32]

^a^Modeling methods for in vivo studies

### Mesenchymal stem cells (MSCs)

#### Bone marrow-derived mesenchymal stem cells

MSCs are ASCs isolated from multiple stromal tissues including bone marrow, umbilical cord, adipose tissue, and others. Bone marrow-derived mesenchymal stem cells (BM-MSCs), as indicated by its name, are MSCs derived from bone marrow and are capable to differentiate into multiple cell types. In rat models of NEC, intraperitoneal injection of BM-MSCs reduces NEC incidences and improves the body weight and clinical sickness score, as well as the histopathological changes by homing to the injured sites [21, 24]. Referring to the means of BM-MSC administration in NEC, intraperitoneal injection was first reported. However, compared to intraperitoneal administration, the intravenous administration of BM-MSCs at the same dose in rats might more significantly increase stem cell engraftments into intestines, with a lower incidence of NEC and better improvement in gut barrier function [14].

In order to enhance the therapeutic effect of BM-MSCs, co-administration with biologically active molecules may achieve synergistic effects. HB-EGF, which is secreted by macrophage-like cells and identified to protect ISCs, has been successfully used in NEC treatment. The combination of HB-EGF and BM-MSC administration intravenously in rat models of NEC leads to the best improvement in intestinal barrier function and survival compared to HB-EGF or BM-MSC administrated alone [14].

BM-MSCs are also proved as tremendous storage of cytokines and growth factors. The secretion of IL-6, VEGF, and hepatocyte growth factor (HGF), which increase the viability and proliferation of intestinal cells, by BM-MSCs under hypoxia mimicking NEC has been confirmed [33]. However, direct application of BM-MSC-conditioned media lacks therapeutic potency, probably due to insufficient release and subsequent degradation. In contrast, the application of prolyl hydroxylase 2 (PHD2)-silenced BM-MSC-conditioned media achieves therapeutic effects in NEC rats [25]. Mechanistically, the BM-MSC-mediated paracrine effects on relieving NEC are contributed by nuclear factor-κB (NF-κB) signaling, and the silencing of PHD2 in BM-MSCs enhances NF-κB activation, which further increases the paracrine release of insulin-like growth factor (IGF)-1 and transforming growth factor-beta 2 (TGF-β2) to reduce intestinal injury [25].

Several failures have been reported referring to the use of BM-MSCs in animal models. Zani et al. attempt to intravenously inject BM-MSCs into NEC rats; however, they do not demonstrate improved survival compared to rats injected with PBS [26]. Fetus suffering from hypoxia-ischemia is under high risk of intestinal injury and developing NEC. Preterm fetal sheep exposed to umbilical cord occlusion, which induces hypoxic-ischemic events, receive BM-MSC administration intravenously but do not show attenuation in intestinal injury [22]. These frustrating results inspire researchers to explore the role of other types of stem cells in NEC therapy.

#### Amniotic fluid-derived mesenchymal stem cells

Since amniotic fluid also contains plenty of stem cells and is conveniently collected during amniocentesis, researchers have focused on amniotic fluid-derived mesenchymal stem cells (AF-MSCs). Good et al. first show that amniotic fluid microinjection into the fetal intestine reduces the severity of NEC in mice models, indicating that amniotic fluid, presumably the AF-MSCs contained inside, can enhance the healing of NEC [26, 34]. In a preterm porcine NEC model, AF administration during both the total parenteral nutrition and enteral nutrition periods increases the gain of body weight and improves the NEC scores [35]. Intraperitoneal injection of AF-MSCs is further performed, and macroscopic gut damage, intestinal function, bowel inflammation, as well as enterocyte proliferation in NEC rats are improved [26]. The improved survival and enhanced repair by AF-MSCs are primarily achieved by paracrine manner, more specifically, the stromal cells expressing cyclooxygenase 2 (COX-2), rather than direct repopulation of injured cells [26]. In clinical practice, neonates with NEC often develop ascites due to fluid retention, which is also a feature in the modified Bell’s NEC staging criteria [36]. AF-MSCs by intraperitoneal injection are shown to significantly decrease fluid retention and the incidence of ascites in NEC rats [27]. The epithelial regeneration and activation of ISCs are rescued by AF-MSCs administered intraperitoneally in NEC mice via activation of the Wnt signaling [28].

#### Umbilical cord-derived mesenchymal stem cells

Similar to amniotic fluid, umbilical blood is rich in stem and progenitor cells, and umbilical cord-derived mesenchymal stem cells (UC-MSCs) are easily accessible with low immunogenicity [37]. The intraperitoneal injection of UC-MSCs exerts beneficial effects in protecting intestines from injury in experimental NEC [29]. Paracrine release of hydrogen sulfide (H_2_S), which confers cytoprotective, antioxidant, and anti-inflammatory functions at low levels, is increased in UC-MSCs under hypoxic conditions, and contributes to the protection of intestine.

#### Other types of mesenchymal stem cells

The research on other types of MSCs, for example, MSCs derived from ESCs or iPSCs, is still insufficient in the field of NEC treatment. Ethical limitations exist for ESCs since it involves the destruction of human embryos, and there are fewer ethical concerns associated with iPSCs because they are directly reprogrammed from adult cells [38]. Recently, Kagia et al. compare the intraperitoneal administration of BM-MSCs, UC-MSCs, ESC-MSCs, and iPSC-MSCs in a chemically induced acute enterocolitis model, resembling NEC histopathologically [39]. In this model, the prolongation of survival in mice by BM-MSC or UC-MSC injection is greater than that by ESC-MSC or iPSC-MSC injection [39]. Furthermore, the clinical and histopathological improvement is only seen in mice under BM-MSC and UC-MSC treatment [39].

### Neural stem cells (NSCs)

The enteric nervous system (ENS), which is able to control gastrointestinal function without input from the brain or spinal cord, is the largest and most complex division of the peripheral nervous system [40]. Newborns have an immature ENS, which is vulnerable to injury and may predispose them to NEC. Newborns with NEC demonstrate a noticeable reduction in neuronal and glial cells within myenteric plexus and external submucosal plexus. NSCs are responsible for the repair and renewal of neurons in ENS, and therefore draw researchers’ attention into this field [41].

Zhou et al. first report the use of NSC injection for NEC therapy, and rat pups receiving NSC transplantation demonstrate increased enteric nervous system integrity, stem cell differentiation, intestinal transit, and survival [30]. NSC transplantation in a mouse model also shows improved gut barrier function and intestinal motility, with decreased NEC incidence [31]. Similar to the beneficial effects of HB-EGF on BM-MSC administration, HB-EGF enhances the proliferation and decreases the apoptosis of NSCs in vitro, and the simultaneous administration of HB-EGF and NSCs in vivo results in decreased intestinal injury scores and improved gut barrier function as well as increased motility by the protection of neurons [31]. HB-GEF also increases NSC differentiation and the expression of neuronal nitric oxide synthase (nNOS), which enhances NO production and protects neurons from degeneration or damage [32].

For clinical practice, the acquisition of NSCs from fetal guts is not practical, and therefore NSCs collected from amniotic fluid can be an alternative for NEC therapy [30, 42]. With the use of amniotic fluid-derived NSCs (AF-NSCs), the incidence and severity of NEC in rats are significantly reduced, and the reduction in NEC incidence were not significantly different compared to that in the enteric NSC (E-NSC) treatment group, BM-MSC treatment group or AF-MSC treatment group, indicating that different types of stem cells might be equivalently effective for NEC treatment under certain biological conditions [24]. Subsequently, the effects of BM-MSCs, AF-MSCs, AF-NSCs, and E-NSCs in improving gut barrier function are evaluated. In spite of the results that the four types of stem cells demonstrate no difference in the capacity to decrease intestinal permeability and improve gut barrier functions, amniotic fluid-derived stem cells are preferred for use because they are easily acquired and cultured [23].

## Exosomes in necrotizing enterocolitis therapy (Table 3)

Although an increasing number of studies have provided evidence that stem cell treatment is beneficial for NEC, several concerns are raised in terms of stem cell therapy. Stem cells can potentially and unexpectedly trigger the immune response. In addition, the acquisition and culture of stem cells are challenging and may bring about ethical issues [11]. Therefore, a potent therapeutic strategy without the direct involvement of stem cells is needed. Inspired by the fact that exosomes with considerable therapeutic efficacy can be derived from most types of cells, including stem cells, researchers consider exosomes as attractive candidates for the NEC therapy.

**Table 3:** Functions and applications of exosomes in NEC

Exosome type	Exosome origin	Exosome isolation	Exosome concentration	Exosome administration	Model	Modeling method^a^	Main findings	Mechanism	Reference
AF-MSC-Ex	Rat	ExoQuick reagent	/	Intraperitoneal injection	In vitro In vivo Ex vivo	Mouse pup with formula, exposure to hypoxia for 4 days and oral LPS injection (4 mg/kg for 2 days)	AF-MSC-Exs increase cellular proliferation, reduce inflammation, and regenerate a normal epithelium. AF-MSC-Exs attenuate NEC intestinal injury via activating the Wnt signaling pathway.	Wnt/β-catenin (ISCs)	[28]
BM-MSC-Ex	Mouse	Serial centrifugation (in vitro) PureExo Exosome Isolation kit (in vivo)	~ 2.5 ×10^9^ exosomes/50 μL	Intraperitoneal injection	In vitro In vivo	Mouse pup with formula, hypoxia (100% N_2_, 1.5 min, bid for 4 days) and hypothermia (4 °C, 10 min, bid for 4 days)	BM-MSC-Exs increase wound healing in vitro. BM-MSC-Exs significantly lower gut permeability and the incidence of NEC in vivo.	/	[43]
AF-MSC-Ex	Rat	Ultra-centrifugation	4 × 10^8^ exosomes/50 μL	Intraperitoneal injection	In vivo	Rat pups with formula, hypoxia (100% N_2_, 1.5 min, tid for 4 days) and hypothermia (4 °C, 10 min, tid for 4 days)	BM-MSC-Exs, AF-MSC-Exs, amniotic fluid-derived NSC-Exs and enteric NSC-Exs demonstrate equivalent reductions in NEC incidence. Stem cell-derived exosomes are equivalent to stem cells in NEC therapy.	/	[44]
BM-MSC-Ex									
NSC-Ex (amniotic fluid-derived)									
NSC-Ex (enteric)									
HM-Ex	Human	Ultra-centrifugation	0–10 μg	/	In vitro	/	HM-Exs reduce oxidative stress-related injury on intestinal epithelial cells.	/	[45]
HM-Ex	Human	Serial centrifugation	0.1 μg/μL	/	Ex vivo	/	HM-Exs derived from colostrum, transitional or mature human milk prevent inflammatory injury. HM-Exs derived from colostrum are most effective in decreasing inflammatory cytokine.	/	[46]
HM-Ex	Human	Ultra-centrifugation	200 μg/mL	Gavage	In vivo	Rat pups with formula and hypoxia (5% O_2_, 75% N_2_, 5 min, bid for 4 days)	HM-Exs promote the proliferation and migration of intestinal epithelial cells both in vitro and in vivo. Peptidomic differences between preterm and term milk exosomes are revealed.	/	[47]
HM-Ex	Human	Ultra-centrifugation	0–1 × 10^8^ exosomes/100 μL	Intraperitoneal injection Gavage	In vitro In vivo	Rat pups with formula, hypoxia (< 1.5% O_2_, 1.5 min, tid for 4 days), hypothermia (4 °C, 10 min, tid for 4 days) and LPS (2 mg/kg, day 1)	HM-Exs increase the proliferation and decrease the apoptosis of intestinal epithelial cells. HM-Exs administered intraperitoneally or enterally decrease NEC incidence. HM-Ex enteral administration has better effects.	/	[48]
HM-Ex	Human	Ultra-centrifugation	1.15–1.19 g/mL	Gavage	Ex vivo In vivo	Mouse pups with formula, hypoxia (5% O_2_, 10 min, tid for 5 days) and LPS (4 mg/kg, qd for 5 days)	HM-Exs reduce inflammation and improve mucus production in vivo. HM-Exs decrease inflammation in hypoxia and LPS-treated intestinal organoids. Pasteurized HM-Exs are as effective as raw HM-Exs.	/	[49]
HM-Ex	Human	ExoQuick reagent	0.5 mg/mL	/	In vitro	/	HM-Exs upregulate Wnt/β-catenin signaling in ISCs and increase cell viability under H_2_O_2_ exposure compared to the control group.	Wnt/β-catenin (ISCs)	[50]
PM-Ex	Pig	Ultra-centrifugation	0.037 mg/μL	Gavage	In vitro In vivo	Mouse pups with LPS (7.5 mg/kg, qd for 7 days)	PM-Exs inhibit intestinal epithelial cell apoptosis and decrease TLR4/NF-κB signaling through miRNAs in vitro. PM-Exs prevent LPS-induced intestinal injury and inflammation in vivo.	miRNAs (miR-4334, -219, -338)	[51]
BovM-Ex	Cow	Ultra-centrifugation	1 μg/μL	Gavage	In vitro In vivo	Mouse pups with formula, hypoxia (5% O_2_, 10 min, tid for 5 days) and LPS (4 mg/kg, day 6 and 7)	BovM-Exs promote goblet cell and endoplasmic reticulum chaperone protein expression both in vitro and in vivo, which increases mucus production and protect the intestine.	TFF3, MUC2 (goblet cell), and GRP94 (endoplasmic reticulum)	[52]

^a^Modeling methods for in vivo studies

### Stem cell-derived exosomes

As described above, stem cells decrease the incidence of NEC in animal models, mainly by a paracrine manner, and therefore the application of stem cell-derived exosomes (SC-Exs), as bioactive factors secreted by stem cells, achieves similar or better protective effects [44]. Rager et al. isolate exosomes from BM-MSCs, and the exposure to BM-MSC-derived exosomes (BM-MSC-Exs) enhances intestinal epithelial cell wound healing in vitro, while the exposure to BM-MSC-conditioned media without exosomes abrogates the wound healing rate [43]. Intraperitoneal injection of BM-MSC-Exs preserves the gut barrier integrity, and reduces the incidence and severity of NEC in rats [43, 44].

Exosomes derived from other types of stem cells, including AF-MSCs, AF-NSCs, and E-NSCs are further compared to evaluate their therapeutic effects. It is shown that BM-MSC-Exs, AF-MSC-derived exosomes (AF-MSC-Exs), AF-NSC-derived exosomes (AF-NSC-Exs), and E-NSC-derived exosomes (E-NSC-Exs) have equivalent effects of improving gut barrier function and reducing NEC incidences in rats [44]. Mechanistically, AF-MSC-Exs have been identified to improve NEC-induced intestinal regeneration by the Wnt signaling [28].

### Human breast milk-derived exosomes

Exosomes are not only released by stem cells, but also present in body fluids, for instance, breast milk. Breast milk is naturally rich in exosomes and can be obtained from the lactating mother or all lactating women, which can be a promising strategy with safety and cost-effectiveness. Compared to mature milk, early milk such as colostrum contains a higher number of exosomes [53]. The onset of NEC is during the 2–3 weeks of life when the colostrum is no longer available, indicating the reduction of bioactive molecules in breast milk might contribute to the NEC development [52].

Martin et al. first show that human breast milk-derived exosome (HM-Ex) administration protects cells from oxidative stress-induced intestinal cell injury [45]. Despite the differences in species, human exosomes are able to protect the intestinal epithelial cells of rat [45]. The effect of HM-Ex on treating NEC is further evaluated in the ex vivo organoid model, which mimics the natural microenvironment of the gut [46, 49]. In addition, researchers try to figure out the impact of lactation period on the therapeutic effects of HM-Exs. Administration of colostrum (day 1–5 postpartum), transitional (day 6–14 postpartum) and mature (day > 15 postpartum) HM-Exs all reduce inflammatory injury in the ex vivo model [46]. Among the three types of exosomes, colostrum-derived exosomes have the best capacity to hamper pro-inflammatory responses, indicating that colostrum is the optimal source of HM-Exs [46]. Moreover, in vivo NEC models are constructed, and the intraperitoneal or enteral administration of HM-Exs decreases NEC incidences in rat pups [47, 48]. Pathological examination of rat intestines reveals that HM-Exs protect the villous integrity from injury and restore cellular proliferation [47].

Milk exosomes exhibit ability to traverse human intestinal epithelial barrier and thus have potential to be administered enterally [54]. HM-Exs administered by the enteral route (p.o.) result in further reduction of NEC incidences compared to those administered intraperitoneally, which indicates that the enteral route is a better option for HM-Ex administration [48]. The mechanisms by which HM-Exs reduce NEC incidences are partially contributed by activation of the Wnt/β-catenin signaling pathway, which increases ISC viability and protects cells from oxidative stress [50].

In clinical practice, Holder pasteurization (62.5 °C for 30 min) is used to guarantee the microbiological safety of donor breast milk; nevertheless, this process may disrupt the benefits brought by HM-Exs [55]. Recently, it is confirmed that both raw and pasteurized HM-Exs decrease the inflammation in ex vivo and in vivo NEC models, and the pasteurized HM-Exs are as effective as the raw HM-Exs [49]. These inspiring preclinical results strongly support the application of HM-Exs clinically.

### Other mammals’ breast milk-derived exosomes

Except for breast milk-derived exosomes isolated from human, exosomes can also be isolated from other mammals, such as murine and bovine. Li et al. first identify that rat breast milk-derived exosomes (RM-Exs) increase the viability and proliferation of intestinal epithelial cells, as well as stem cell activity, which are promising for treating NEC [52]. Porcine milk-derived exosomes (PM-Exs) gavage suppresses intestinal damage and LPS-induced inflammation in mice [51]. In vitro, LPS-induced apoptosis and inflammation of intestinal epithelial cells are decreased by PM-Exs [51]. The cytoprotective effects are induced by TLR4/NF-κB signaling and apoptotic pathway suppression mediated by exosome microRNAs (miRNAs), suggesting that miRNA-enriched exosomes can be used as a novel preventative strategy for NEC [51, 56].

Although RM-Exs and PM-Exs are restricted to experimental use due to limitations on their sources, bovine milk-derived exosomes (BovM-Exs) possess great potential to be used in clinical practice, especially when sufficient human breast milk is not available. To mimic the normal fashion of nutrient administration in neonates, BovM-Exs are added to formula and given to NEC mice through gavage feeding. The administration of BovM-Exs increases goblet cell expression markers trefoil factor 3 (TFF3) and mucin 2 (MUC2), as well as the expression of endoplasmic reticulum chaperone protein glucose-regulated protein 94 (GRP94) both in vitro and in vivo, which enhances mucus production and protects gut barrier function [52, 57, 58].

## Conclusions and future directions

NEC is a notorious disease threatening the lives of neonates and brings enormous health and economic burden to the society. Early and effective management of necrotizing enterocolitis can not only alleviates intestinal injury, but also reduce complications, for example, brain injury and cognitive impairment [59]. Therefore, novel therapeutic approaches for NEC are needed.

Stem cell research on NEC has progressed tremendously in the past decade, demonstrating that stem cells are effective in reducing inflammation, improving gut barrier function and maintaining intestinal function. Among the three main types of stem cells (ISCs, MSCs, NSCs) that are focused on by researchers, ISCs are well elucidated for their physiological roles, while MSCs and NSCs are well described for their therapeutic effects after administration. To be specific, the therapeutic effect of BM-MSCs for NEC remains controversial, while AF-MSCs are a relatively more appropriate choice for potential clinical application, because they are more easily obtainable and may offer superior beneficial effects than other types of MSCs [26].

Compared to stem cell transplantation, administration of exosomes possesses distinct superiority. Compared to exosomes derived from stem cells, exosomes derived from human milk can be more easily obtained and orally administrated. Exosomes derived from colostrum demonstrate the best effects, and the pasteurization of human milk does not attenuate their effects [55]. In addition to human milk, bovine milk serves as another source of exosomes, and the therapeutic effects of BovM-Exs to NEC have been confirmed in vivo. Therefore, milk-derived exosomes are a safe, convenient, and effective strategy for NEC therapy.

Optimization of therapeutic effects can be achieved by several strategies in stem cell and exosome therapy. Stem cell modifications, including genetic modification and preconditioning modification, have been applied to improve the therapeutic properties. Genetic modification through constructed gene cassettes, preconditioning modifications by biological factors both improve the migration, adhesion, and survival, as well as reduce the premature senescence of stem cells [60]. Integrating stem cells with natural or synthetic biomaterial scaffolds, which enhance cell viability, differentiation and therapeutic efficacy, is effective for stem cell transplantation [61].

Advances in nanoengineering show great promise for the targeted delivery of exosomes towards the injured intestine, optimizing the therapeutic effects of exosomes. Anchor peptides with corresponding targets on exosomes enable direct loading of exosomes [62]. Magnetically modified exosomes are efficient for tissue-specific delivery, whereas they might be toxic due to magnetic nanoparticles [63]. Aptamer-mediated exosome delivery demonstrates easy operation, enhanced efficacy, and high cost-effectiveness, which have become a superior choice of targeted therapy [64]. Nevertheless, research on engineered exosomes is in its infancy, and no evidence on the application of nanoengineered exosomes in the NEC therapy has been reported. Because targeted exosome delivery is fast-developing and promising, their application in the treatment of NEC should be brought to the forefront.

**Fig. 1:**
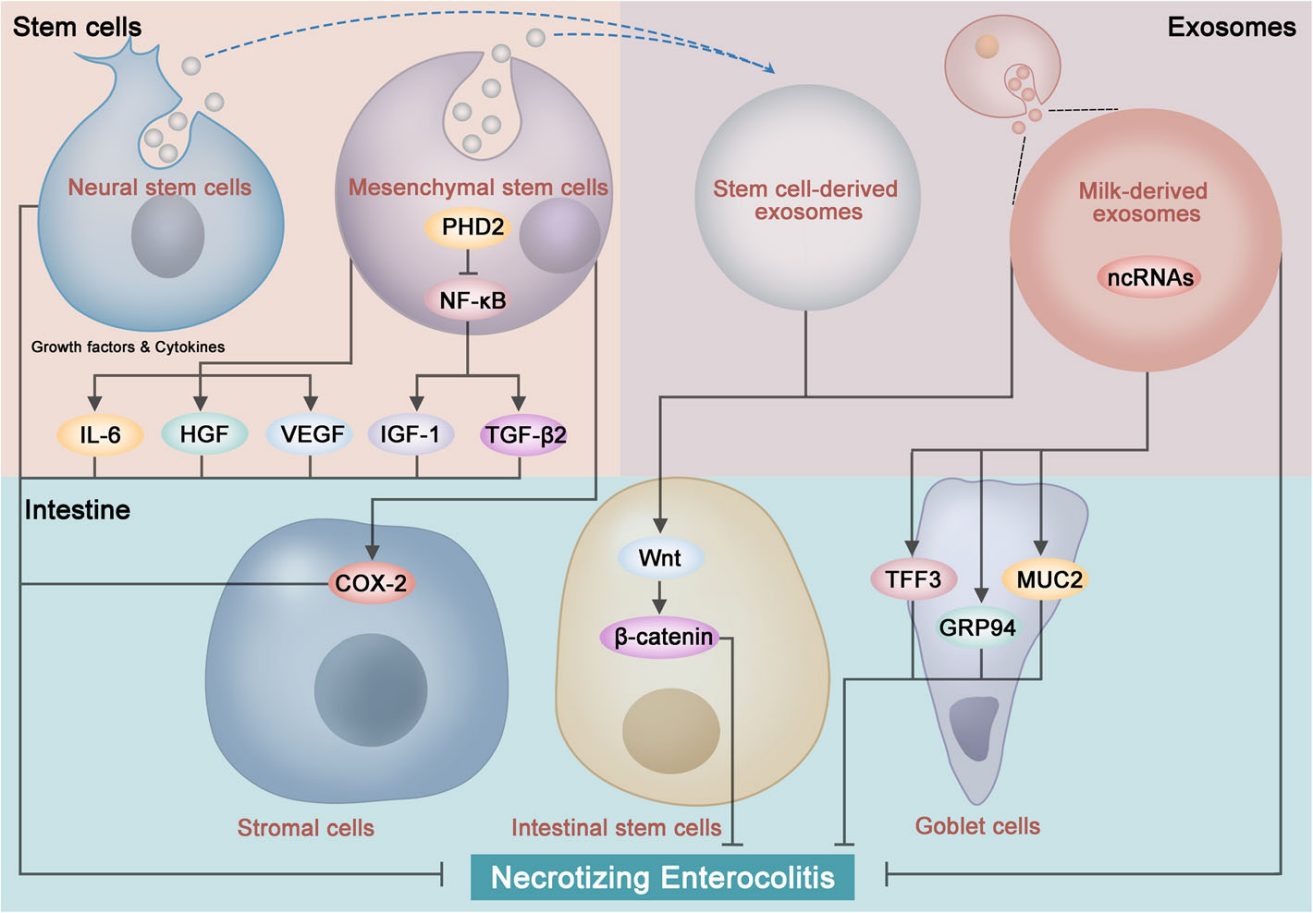
Functions and mechanisms of stem cells and exosomes in necrotizing enterocolitis (NEC). Stem cells and exosomes exert beneficial effects to NEC via various signaling pathways. Mesenchymal stem cells (MSCs) secrete cytokines and growth factors including interleukin (IL)-6, vascular endothelial growth factor (VEGF), and hepatocyte growth factor (HGF). Downregulation of prolyl hydroxylase 2 (PHD2) activates the nuclear factor-κB (NF-κB) signaling in MSCs, which increases the paracrine release of insulin-like growth factor (IGF)-1 and transforming growth factor-beta 2 (TGF-β2). MSCs increase the expression of cyclooxygenase 2 (COX-2) in stromal cells by the paracrine manner. Exosome (Exs) contains non-coding RNAs (ncRNAs) beneficial to NEC injury. Milk-derived Exs increase goblet cell expression markers trefoil factor 3 (TFF3) and mucin 2 (MUC2), as well as the expression of endoplasmic reticulum chaperone protein glucose-regulated protein 94 (GRP94). Both MSCs and MSC-derived Exs can activate the Wnt/β-catenin signaling pathway, which increases ISC viability and intestinal regeneration

To achieve the best therapeutic effects, understanding the molecular pathways of how stem cells and exosomes function in relieving NEC is essential (Fig. 1). Overlapping mechanisms currently identified in NEC pathogenesis and stem cell/exosome therapy strengthens the rationality of treatment with stem cells and exosomes. Stem cells exert their effects through a paracrine manner, including growth factors, cytokines, and exosomes. Exosomes can further modulate the signaling pathways in the cells of intestines, for example, goblet cells and ISCs, which are crucial for gut barrier function and injury repair, partially via the non-coding RNAs (ncRNAs) inside. Current evidence indicates that stem cells and exosomes improve microcirculation, modulate immune responses, and alleviate inflammation [65, 66]. The impaired Wnt signaling and ISC-mediated intestinal regeneration in the pathogenesis of NEC can be restored by both stem cells and exosomes. Despite these findings, the mechanisms remain largely unknown and can be further explored in the following aspects: (1) the most essential cytokines or growth factors can be identified; (2) more specific pathways associated with the therapeutic effects of stem cells and exosomes, particularly for those overlapping with the pathogenesis of NEC, can be explored; (3) the downstream pathways can be further confirmed in vivo; (4) omics research, including genomics, epigenomics, transcriptomics, proteomics, and metabolomics, can be performed; and (5) effects and mechanisms of stem cells and exosomes in improving complications of NEC can be assessed.

**Fig. 2:**
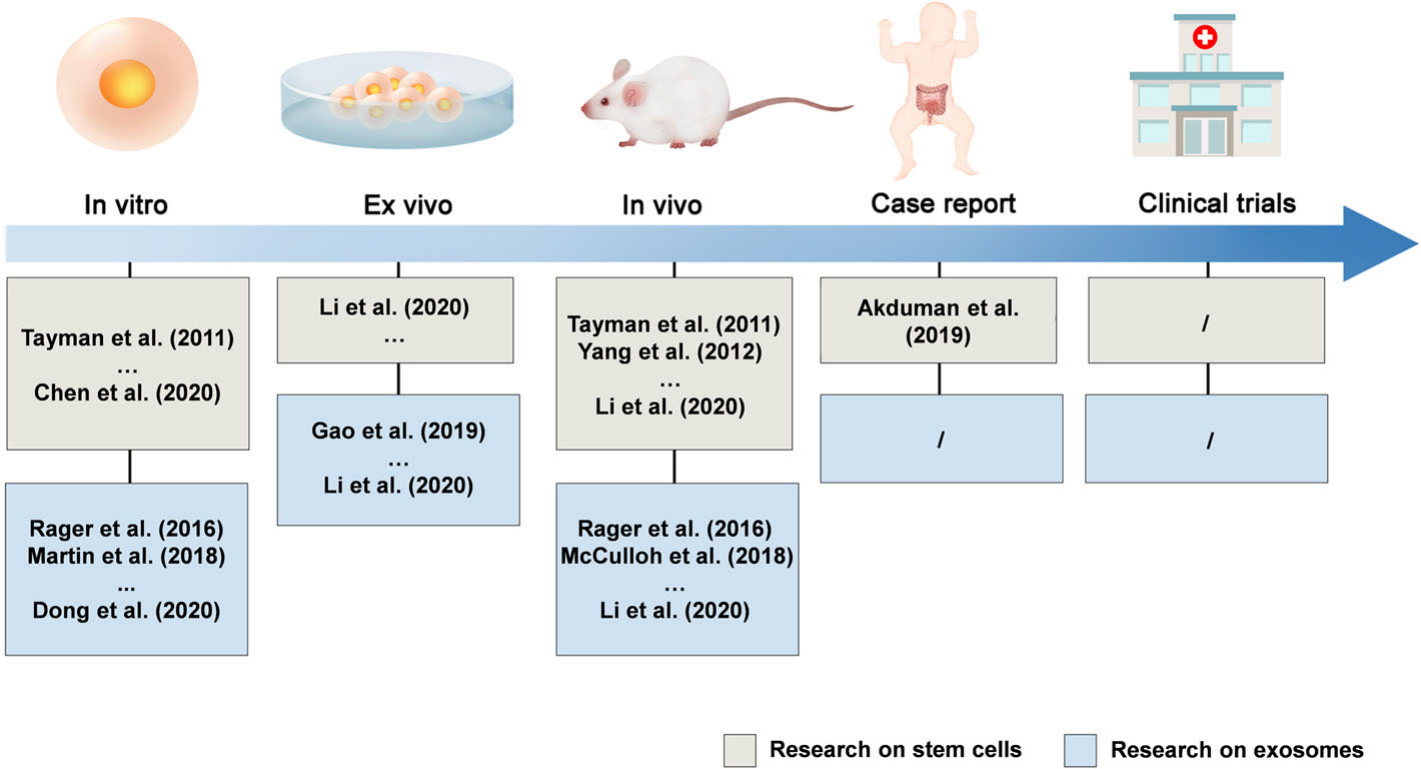
Research progress in stem cells and exosomes for the necrotizing enterocolitis (NEC) treatment. Evidence has indicated the successful use of stem cells and exosomes in treating NEC. Rigorous clinical trials are needed for the application of stem cells and exosomes in the NEC therapy

Recently, the application of stem cells in the NEC therapy clinically was reported. A 22-day old male baby received intravenous injection of UC-MSCs on the 4th day of surgery, and jejunostomy on the 46th day after surgery demonstrated remarkable improvement in intestinal blood supply, without the development of short bowel syndrome [67]. The case report is encouraging; however, the process of exosome isolation and purification remains to be improved. Rigorous clinical trials are needed to verify the safety, efficacy, and optimal usage of stem cells and exosomes for the NEC therapy (Fig. 2).

In conclusion, stem cells and exosomes, which have been demonstrated promising effects in both experimental and preclinical studies, are promising candidates for the NEC therapy. Further elucidation of mechanisms, improvements in preparation, bioengineering, and application, as well as rigorous clinical trials, will facilitate the application of stem cells and exosomes as novel strategies for pediatric diseases.

## Data Availability

Not applicable.

## Abbreviations

AF-MSC: Amniotic fluid-derived mesenchymal stem cell
AF-NSC: Amniotic fluid-derived neutral stem cell
ASC: Adult stem cell
BM-MSC: Bone marrow-derived mesenchymal stem cell
BovM-Ex: Bovine milk-derived exosome
COX-2: Cyclooxygenase 2
E-NSC: Enteric neural stem cell
EGF: Epidermal growth factor
ENS: Enteric nervous system
ESC: Embryonic stem cell
EV: Extracellular vesicle
Exs: Exosomes
GRP94: Glucose-regulated protein 94
H2S: Hydrogen sulfide
HB-EGF: Heparin-binding epidermal growth factor-like growth factor
HGF: Hepatocyte growth factor
HM-Ex: Human breast milk-derived exosome
IGF: Insulin-like growth factor
IL: Interleukin
iPSC: Induced pluripotent stem cell
ISC: Intestinal stem cell
LPS: Lipopolysaccharide
miRNA: MicroRNA
MSC: Mesenchymal stem cell
MUC2: Mucin 2
ncRNA: Non-coding RNA
NEC: Necrotizing enterocolitis
NF-κB: Nuclear factor-κB
nNOS: Neuronal nitric oxide synthase
NSC: Neural stem cell
PHD2: Prolyl hydroxylase 2
PM-Ex: Porcine milk-derived exosome
RM-Ex: Rat breast milk-derived exosome
TFF3: Trefoil factor 3
TGF-β2: Transforming growth factor-beta 2
TLR: Toll-like receptor
UC-MSC: Umbilical cord-derived mesenchymal stem cell
